# Partial MHC/Neuroantigen Peptide Constructs: A Potential Neuroimmune-Based Treatment for Methamphetamine Addiction

**DOI:** 10.1371/journal.pone.0056306

**Published:** 2013-02-27

**Authors:** Jennifer M. Loftis, Clare J. Wilhelm, Arthur A. Vandenbark, Marilyn Huckans

**Affiliations:** 1 Research and Development, Portland VA Medical Center, Portland, Oregon, United States of America; 2 Behavioral Health & Clinical Neurosciences Division, Portland VA Medical Center, Portland, Oregon, United States of America; 3 Department of Psychiatry, School of Medicine, Oregon Health and Science University, Portland, Oregon, United States of America; 4 Department of Neurology, School of Medicine, Oregon Health and Science University, Portland, Oregon, United States of America; 5 Department of Molecular Microbiology and Immunology, School of Medicine, Oregon Health and Science University, Portland, Oregon, United States of America; University of Medicine & Dentistry of NJ - New Jersey Medical School, United States of America

## Abstract

Relapse rates following current methamphetamine abuse treatments are very high (∼40–60%), and the neuropsychiatric impairments (*e.g.*, cognitive deficits, mood disorders) that arise and persist during remission from methamphetamine addiction likely contribute to these high relapse rates. Pharmacotherapeutic development of medications to treat addiction has focused on neurotransmitter systems with only limited success, and there are no Food and Drug Administration approved pharmacotherapies for methamphetamine addiction. A growing literature shows that methamphetamine alters peripheral and central immune functions and that immune factors such as cytokines, chemokines, and adhesion molecules play a role in the development and persistence of methamphetamine induced neuronal injury and neuropsychiatric impairments. The objective of this study was to evaluate the efficacy of a new immunotherapy, partial MHC/neuroantigen peptide construct (RTL551; pI-A^b^/mMOG-35-55), in treating learning and memory impairments induced by repeated methamphetamine exposure. C57BL/6J mice were exposed to two different methamphetamine treatment regimens (using repeated doses of 4 mg/kg or 10 mg/kg, s.c.). Cognitive performance was assessed using the Morris water maze and CNS cytokine levels were measured by multiplex assay. Immunotherapy with RTL551 improved the memory impairments induced by repeated methamphetamine exposure in both mouse models of chronic methamphetamine addiction. Treatment with RTL551 also attenuated the methamphetamine induced increases in hypothalamic interleukin-2 (IL-2) levels. Collectively, these initial results indicate that neuroimmune targeted therapies, and specifically RTL551, may have potential as treatments for methamphetamine-induced neuropsychiatric impairments.

## Introduction

Methamphetamine use causes long-term neuropsychiatric impairments (*e.g.*, cognitive deficits, anxiety, and depression) that make addiction to this drug extremely challenging to treat [1], [2]. A third to half or more of methamphetamine dependent adults experience persistent mood, anxiety, and other psychiatric disorders up to three years or longer into remission [3], [4], [5], [6], [7]. Relapse rates following current substance abuse treatments are very high (∼40–60%) [8], and the neuropsychiatric impairments that arise and persist during remission from methamphetamine addiction likely contribute to these high relapse rates [5]. Pharmacotherapeutic development of medications to treat addiction has centered on neurotransmitter systems with only limited success, and there are no Food and Drug.

Administration (FDA) approved pharmacotherapies for methamphetamine addiction. Alternative treatment strategies are desperately needed.

Methamphetamine dependence is associated with long-term structural damage to regions of the brain that control cognitive and psychiatric function [9], [10], [11]. A growing literature demonstrates that methamphetamine alters peripheral and central immune functions [12]–[18] and that immune factors such as cytokines, chemokines, and adhesion molecules play a role in the development of methamphetamine induced neuronal injury and neuropsychiatric impairments [19], [20], [21]. The cellular source for these immune factors is currently under investigation, but glial cells are thought to play a significant role. Recent studies show that methamphetamine increases the expression of pro-inflammatory cytokines in astrocytes [18] and microglia [22], [23]. These methamphetamine induced neuroimmune and neuropsychiatric impairments parallel immunological responses to other brain insults such as multiple sclerosis, stroke, and other drugs of abuse (*e.g.*, compromised blood brain barrier resulting in increased permeability, increased activation of microglia and astrocytes producing pro-inflammatory cytokines, and damaged myelin/white matter tracks [9], [24]–[36]). Collectively, these lines of evidence support the theory that methamphetamine induced dysregulation of immune function and immune factor expression play a key role in the development and maintenance of methamphetamine induced neuronal changes and neuropsychiatric dysfunction. Within this delicate balance of immune factors lies significant potential for intervention through targeted immunotherapies [37].

Because it has been previously shown that a partial MHC/neuroantigen peptide construct (RTL551; pI-A^b^/mMOG-35-55) effectively reduces the inflammatory and behavioral effects of experimental models of multiple sclerosis and stroke [38], [39], [40], [41], we hypothesized that partial MHCs could also effectively address the neuropsychiatric effects of chronic methamphetamine addiction. The objective of this study was to evaluate the efficacy of multiple injections of RTL551 in treating learning and memory impairments induced by repeated methamphetamine exposure in mice. Experiments were performed using male C57BL/6J mice. Cognitive function was evaluated with the Morris water maze and CNS cytokine expression was measured by multiplex assay. The results of these experiments demonstrated that RTL551 has a treatment effect for memory impairments induced by repeated methamphetamine exposure, as demonstrated in two different mouse models of methamphetamine addiction (*i.e.*, 4 mg/kg/day and 40 mg/kg/day).

## Materials and Methods

### Animals

Male C57BL/6J mice (between 6–12 weeks of age at the beginning of each experiment; approximately 20 g) were obtained from Jackson Laboratories (Bar Harbor, ME, USA). To monitor health status, mice were weighed and observed daily during the course of methamphetamine exposure and drug treatment. *Ethics statement*: This study was carried out in strict accordance with the recommendations in the Guide for the Care and Use of Laboratory Animals of the National Institutes of Health. The Portland VA Medical Center Institutional Animal Care and Use Committee approved all experiments utilizing these mice.

### Drugs

Methamphetamine was obtained from the National Institute on Drug Abuse (NIDA) drug supply program (*i.e.*, NIDA Chemistry & Physiological Systems Research Branch). Methamphetamine was dissolved in 0.9% saline and stored at 4°C. The stock solution was stored for up to 10 days prior to administering doses to mice. Stock solutions were aliquoted as needed on the day of treatment.

The partial major histocompatibility complex (MHC) constructs (pMHCs) were received from Drs. Gregory Burrows and Roberto Meza-Romero of the Oregon Health & Science University, Portland, OR. RTL551 consists of I-A^b^ MHC domains linked to the myelin oligodendrocyte glycoprotein (MOG)-35-55 peptide. The physical properties and mechanisms of action of RTLs (or pMHCs) have been described in detail previously [38], [39], [40], [41], [42]. Recombinant proteins (RTL551) were purified by fast protein liquid chromatography (FPLC) using a Source 30Q anion-exchange column run followed by a Superdex 75 size exclusion column run. Proteins were then dialyzed against 20 mM Tris-Cl buffer at pH 8.5, which removed the urea and allowed refolding of the recombinant proteins. Following dialysis, the proteins were concentrated by ultrafiltration cell through an ultrafiltration membrane with 10,000 molecular weight cut off limit. The final purified products were in 20 mM Tris-Cl buffer, pH 8.5 at a final concentration of 1.0 mg/ml quantified by amino acid analysis. Stock solutions of RTL were stored at −20°C until used and then stored at 4°C thereafter. Dilutions of stock solutions were made prior to administering diluted doses to mice and stored at 4°C.

### Behavioral testing

Mice were evaluated for spatial learning and memory using the Morris water maze (MWM) as previously done [43], with procedures adapted from [44]. The water maze consisted of a round white plastic tub (1.22 m in diameter) filled with water made opaque by the addition of white tempera (non-toxic) paint. Within the tub, a round platform 0.5 m high and 0.13 m in diameter made out of transparent plastic was submerged just below the surface of the water. Animals received two days of visible platform training, followed by three days of hidden platform training. For visible platform trials, a 50 ml conical tube (Becton, Dickinson and Company, Franklin Lakes, NJ) labeled with bright laboratory tape was used to mark the submerged platform, and the platform was placed in each of the four quadrants of the water maze. Animals received three trials (spaced 8–10 min apart) at each visible platform location. Trials were 60 s from the time the animal was placed in the water maze, or until the animal located and climbed atop the platform. Animals that did not find the platform within 60 s were moved to the platform and held there for three seconds. Following the two days of visible platform training, animals received three days of hidden platform training. For these trials, the location of the platform was fixed and the 50 ml conical tube marking the platform removed. Hidden platform training was similar to visible platform training, with animals receiving six trials per day (broken into two sets of three trials with three hours separating each set of trials). Spatial learning and memory was assessed daily via a single probe trial that occurred 60 min after the last hidden platform training trial. The hidden platform was removed prior to the probe trials, and each probe trial lasted for 60 s. An additional probe trial administered 24 hr after the final hidden platform training session was used to assess long-term memory retention. Behavior was monitored using videotracking software [ANY-maze, Stoeling Co., Wood Dale, IL (Investigation of repeated methamphetamine exposure and RTL treatment on cognitive function); HVS Image 2020 Plus Tracking System, HVS Image Ltd., UK (Evaluation of binge methamphetamine exposure and RTL treatment on cognitive function)].

**Figure 1:**
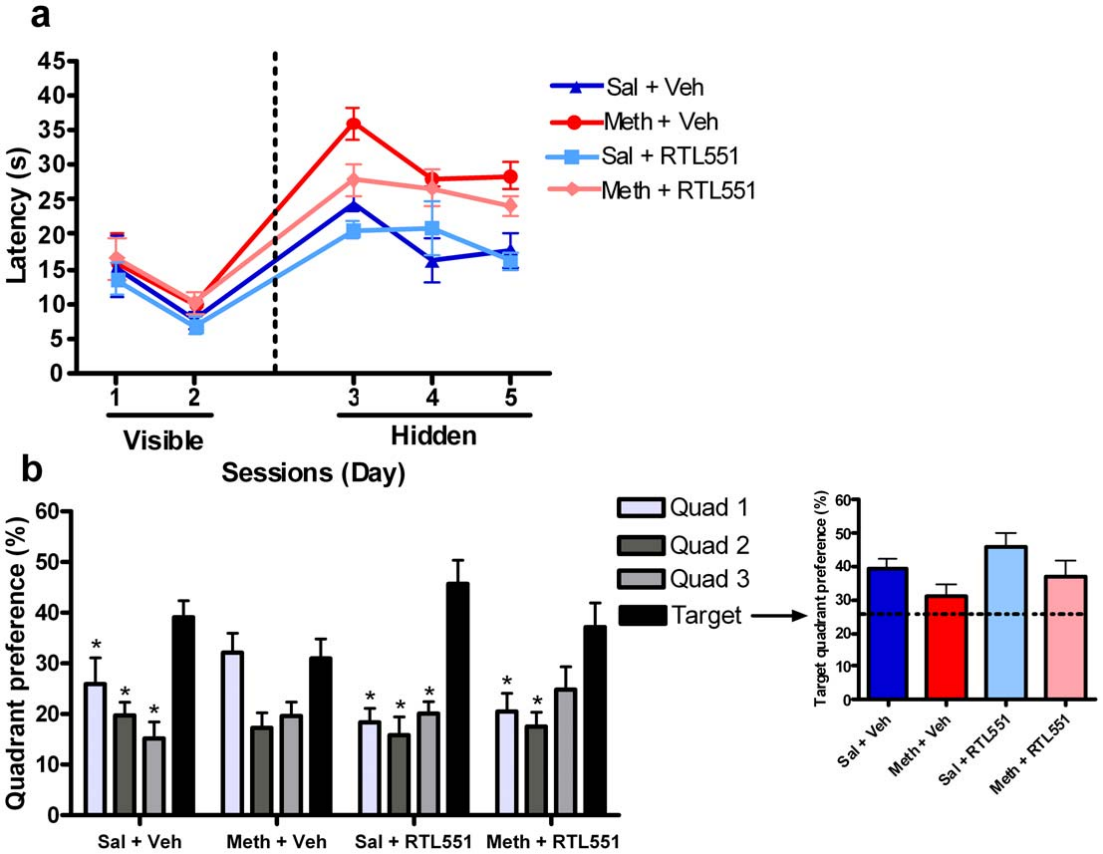
Efficacy of RTL551 over vehicle in the treatment of methamphetamine induced spatial learning and memory impairments (n = 8 per group). (a) Training sessions: time spent finding the visible and hidden platforms. Kruskal-Wallis tests revealed no significant treatment group differences across study groups in terms of the latency to find the platform during the visible platform training sessions [Days 1 and 2; p = 0.39 (Gaussian approximation)]; this demonstrates that the groups were not significantly different in terms of basic motor function and perceptual abilities. Similar analyses also revealed no significant differences across treatment groups in terms of latency to find the platform during the hidden platform sessions [Days 3–5; p = 0.39 (Gaussian approximation)], suggesting that all groups were able to learn the task. (b) Memory test: Quadrant preference during the final 24-hour probe trial. Friedman and Dunn post hoc tests revealed the following differences between the target and the three non-target quadrants: 1) Sal+Veh: Target versus Quadrants 1, 2, and 3 (p<0.05), 2) Sal+RTL551: Target versus Quadrants 1 (p<0.05), 2 (p<0.01), and 3 (p<0.05), and 3) Meth+RTL551: Target versus Quadrants 1, 2, and 3 (p<0.05). Collectively, these results indicate that the Sal treatment groups successfully learned where the hidden platform was located during the probe trial. However, in the Meth+Veh group, the percent time spent in each of the quadrants was not statistically different across quadrants, indicating that methamphetamine had induced significant memory impairments in this group. In the Meth+RTL551 group, mice spent significantly more time in the target quadrant than in two out of the three other quadrants, indicating that RTL had significantly attenuated the methamphetamine induced spatial memory impairments in this group. The inset graph illustrates the differences in preference for the target quadrant across all treatment groups. Statistical analysis found that there was a trend toward a significance difference in preference for the target quadrant [Kruskal-Wallis test, p = 0.10 (Gaussian approximation)]. Preference greater than 25% (dotted black line) suggests that spatial learning was, in part, retained.

**Figure 2:**
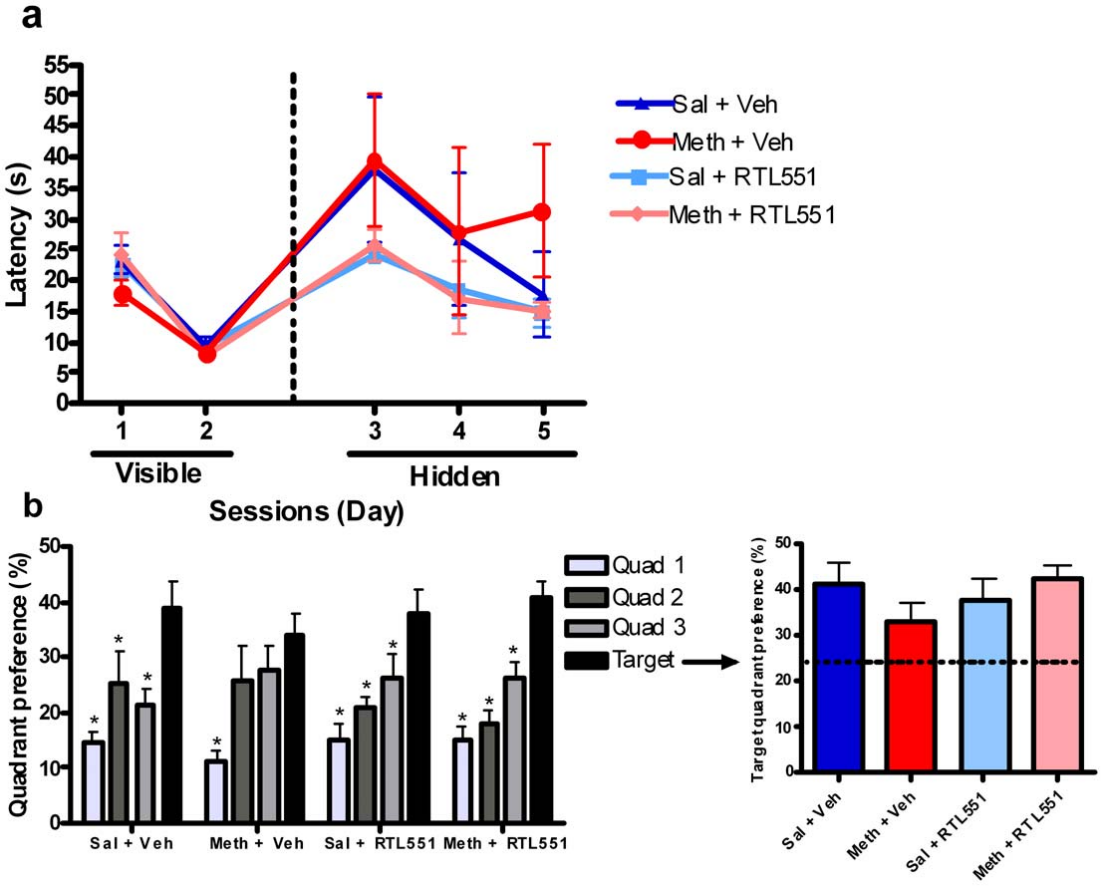
Efficacy of RTL551 over vehicle in the treatment of methamphetamine induced spatial learning and memory impairments (n = 13–14 per group). (a) Training sessions: time spent finding the visible and hidden platforms. Statistical analyses revealed no significant treatment group differences across study groups in terms of the latency to find the platform during the visible platform training sessions [Days 1 and 2; p = 0.39 (Gaussian approximation)]. This demonstrated that the groups were not significantly different in terms of basic motor function and perceptual abilities after treatment. Similar analyses also revealed no significant differences across treatment groups in terms of latency to find the platform during the hidden platform sessions [Days 3–5; p = 0.39 (Gaussian approximation)], suggesting that all groups were able to learn the task. (b) Memory test: Quadrant preference during the Day 5 probe trial (3^rd^ day of hidden platform training). Statistical analyses revealed significant differences between the target and the three non-target quadrants for the following treatment groups: 1) Sal+Veh: Target versus Quadrants 1 (p<0.001), 2 (p<0.01) and 3 (p<0.05), 2) Meth+Veh: Target versus Quadrant 1 (p<0.001), 3) Sal+RTL551: Target versus Quadrants 1 (p<0.05), 2 (p<0.01), and 3 (p<0.01), 4) Meth+RTL551: Target versus Quadrants 1 (p<0.001), 2 (p<0.01) and 3 (p<0.05). In the Meth+Veh group, the percent time spent in each of the quadrants was only statistically different between quadrant 1 and the target quadrant, indicating that methamphetamine had induced significant memory impairments in this group. However, in the Meth+RTL551 group, mice spent significantly more time in the target quadrant than in all three of the other quadrants, indicating that RTL had significantly attenuated the methamphetamine induced spatial memory impairments in this group. The inset graph illustrates the differences in preference for the target quadrant across all treatment groups. When comparing all treatment groups, there was not a statistically significant difference in preference for the target quadrant [Kruskal-Wallis test, p = 0.27 (Gaussian approximation)]. However, exploratory post-hoc analyses found a significant difference between Meth+Veh and Meth+RTL551 treatment groups [Mann-Whitney test, p = 0.034 (Gaussian approximation)].

The primary dependent variable for this task was the amount of time animals spent in the target quadrant (the quadrant that was paired with the hidden platform) during probe trials. The amount of time spent in the target quadrants during probe trials is indicated by quadrant preference (% time) in Figures 1B and 2B. Latency or time to find the platform during the visible and hidden platform training trials was also measured and analyzed (Figs. 1A and 2A).

### Investigation of repeated methamphetamine exposure and RTL treatment on cognitive function

The study design is outlined in Table 1. Male C57BL/6J mice (n = 32) were group housed (n = 4–5 per cage). Mice were randomly assigned to one of four groups: methamphetamine (Meth)-vehicle (Veh), Meth-RTL551, saline (Sal)-Veh, or Sal-RTL551. Animals received methamphetamine (4 mg/kg) or saline once daily [subcutaneously (s.c.)] for 15 consecutive days. During the last 7 days of methamphetamine exposure, animals were also administered (s.c.) RTL551 (0.1 mg/animal s.c.) or Veh (20 mM Tris, 10% w/v dextrose) (Intervention Phase). Mice were weighed daily during drug treatment and behavioral testing to monitor health status.

**Table 1:** Study Design: Investigation of repeated methamphetamine exposure and RTL treatment on cognitive function.

Treatment Group (n)	Drug Exposure Phase (7 Days)	Intervention Phase a (8 Days)	Behavioral Testing Phase a (6 Days)
SAL+Veh (n = 8)	SAL, 0.9%, s.c, once daily	SAL, 0.9%, s.c, once daily; Tris-HCl, pH = 8.5, s.c., once daily	SAL, 0.9%, s.c, once daily; Tris-HCl, pH = 8.5, s.c., once daily
Meth+Veh (n = 8)	Meth, 4 mg/kg, s.c., once daily	Meth, 4 mg/kg, s.c., once daily; Tris-HCl, pH = 8.5, s.c., once daily	Meth, 4 mg/kg, s.c., once daily; Tris-HCl, pH = 8.5, s.c., once daily
SAL+RTL551 (n = 8)	SAL, 0.9%, s.c, once daily	SAL, 0.9%, s.c, once daily; RTL551, 100 µg, s.c., once daily	SAL, 0.9%, s.c, once daily; RTL551, 100 µg, s.c., once daily
Meth+RTL551 (n = 8)	Meth, 4 mg/kg, s.c., once daily	Meth, 4 mg/kg, s.c., once daily; RTL551, 100 µg, s.c., once daily	Meth, 4 mg/kg, s.c., once daily; RTL551, 100 µg, s.c., once daily

a The Intervention and Behavioral Testing Phases overlapped such that MWM training began on the 4^th^ day of the Intervention Phase. To avoid acute intoxication effects at the time of behavioral testing, mice performed water maze testing in the morning, with drug administration later in the day.

Because it is common for human patients with methamphetamine dependence to relapse or continue to use methamphetamine during substance abuse treatment [45], the methamphetamine exposed mouse groups continued to receive methamphetamine during the Intervention Phase. The Intervention Phase was accompanied by a Behavioral Testing Phase during which mice were evaluated for spatial learning and memory with the MWM (*see Behavioral testing*). On the MWM, a lack of preference for the target quadrant (*i.e.*, no significant differences in terms of the time spent in each quadrant across quadrants) was interpreted as impaired spatial learning and memory and analyzed [46], as described under Statistical analysis.

### Evaluation of binge methamphetamine exposure and RTL treatment on cognitive function and CNS cytokine expression

To more closely model the human experience and test the efficacy of RTL in reducing persistent methamphetamine-induced cognitive deficits, we assessed the effects of RTL treatment on cognitive function and cytokine expression when treatment was administered during remission from repeated binge exposure to methamphetamine. This models an ideal treatment strategy in humans where treatment is provided during early remission from addiction. Of note, the cognitive impairments resulting from repeated low dose regimens (*e.g.*, 4 mg/kg for 15 consecutive days) attenuate in mice following the last methamphetamine exposure (*unpublished observations*); thus, a repeated low dose model does not mimic the persistent neuropsychiatric impairments seen in humans during remission from methamphetamine addiction [19] and does not provide a measurable signal to treat during a remission period. The study design for the evaluation of binge methamphetamine exposure and RTL treatment on cognitive function is outlined in Table 2. All mice were singly housed for seven days prior to methamphetamine exposure, and the mice remained housed so until the end of the experiment. Social isolation was introduced to provide the animals with a mild environmental stressor, which parallels the experience of human methamphetamine users. Social isolation also impacts drug and cognitive effects [47], [48].

**Table 2:** Study Design: Evaluation of binge methamphetamine exposure and RTL treatment on cognitive function.

Treatment Group a (n)	Drug Exposure Phase b (14 Days)	Intervention Phase (5 Days)	Behavioral Phase c (6 Days)
SAL+Veh (n = 8)	SAL, 0.9%, s.c, 4× daily	Tris-HCl, pH = 8.5, s.c., once daily	Tris-HCl, pH = 8.5, s.c., once daily
Meth+Veh (n = 8)	Meth, 10 mg/kg, s.c., 4× daily	Tris-HCl, pH = 8.5, s.c., once daily	Tris-HCl, pH = 8.5, s.c., once daily
SAL+RTL551 (n = 8)	SAL, 0.9%, s.c, 4× daily	RTL551, 100 µg, s.c., once daily	RTL551, 100 µg, s.c., once daily
Meth+RTL551 (n = 8)	Meth, 10 mg/kg, s.c., 4× daily	RTL551, 100 µg, s.c., once daily	RTL551, 100 µg, s.c., once daily

a Two cohorts (n = 32 mice per cohort) were used to evaluate binge methamphetamine exposure and RTL treatment on cognitive function.

b The Drug Exposure Phase was preceded by a 7-day period of social isolation for all treatment groups. During the Drug Exposure Phase, each injection was separated by two hours and treatments occurred every other day.

c The Intervention and Behavioral Testing Phases overlapped such that MWM training began on the 1^st^ day of the Intervention Phase.

Two cohorts of mice (n = 32 per cohort) were used to evaluate the effects of binge methamphetamine exposure and RTL treatment on cognitive function. Mice were divided into each of the following four treatment groups: Meth-Veh, Meth-RTL551, Sal-Veh, or Sal-RTL551. Immediately following seven days of isolate housing, mice received repeated daily injections of methamphetamine (10 mg/kg per injection, s.c.), four times per day, with each treatment separated by two hours. Methamphetamine exposure was repeated for seven days, with treatments occurring every other day (*i.e.* methamphetamine exposure occurred on Mon, Wed, Fri, Sun, Tues, Thurs, and Sat). The first day after the methamphetamine treatment was completed, animals began MWM training, and daily treatment with RTL551 (0.1 mg/animal s.c.) or Veh (20 mM Tris, 10% w/v dextrose). RTL treatments were administered on five occasions and followed completion of each day's MWM training.

CNS cytokine expression (cohort 1 only): Four days after completion of the MWM mice were euthanized via CO_2_ asphyxiation (for the determination of cytokine expression). Brains were collected and microdissected on ice; hypothalamus was collected and stored at −80°C. For multiplex determination of cytokine and chemokine expression, brain tissue was prepared based on the methods of Hulse et al., (2004) and as done in our laboratory [49], [50]. Briefly, hypothalamic samples were thawed in cell lysis buffer (Bio-Rad; Hercules, CA), containing protease inhibitor cocktail (Bio-Rad) and 3 µl of a stock solution containing 500 mM phenylmethylsulfonyl fluoride in DMSO (Sigma). The samples were then homogenized (Pellet Pestle motor, Kontes) and centrifuged at 4500× g for 15 min at 4°C. Supernatants were collected and used for multiplex immunoassays. Total protein concentration was determined using BCA (bicinchoninic acid) protein assay kits (Pierce) and absorbance reader (BioRad 680). A multiplex cytokine assay (Mouse Multiplex Cytokine kit, Millipore; Billerica, MA) was conducted to detect interferon-gamma (IFN-γ), IL-10, IL-1β, IL-2, IL-6, monocyte chemoattractant protein-1 (MCP-1 or CCL2), and tumor necrosis factor-alpha (TNF-α) levels.

### Statistical analysis

Mean latency [seconds (s)] to find the platform during each of the visible and hidden platform training sessions (Days 1–5) was summarized for each group, and compared across groups using Kruskal-Wallis tests for the visible and hidden platform sessions. To evaluate memory function, mean percent time spent in each of the four quadrants during the probe trials were summarized for each group, and for each group, the percent time spent in each quadrant was compared across quadrants using Friedman tests (non parametric test for repeated measures), followed by Dunn post hoc tests. Lack of preference for the target quadrant (*i.e.*, no significant differences in terms of the time spent in each quadrant) was interpreted as impaired spatial learning and memory, as has been done previously [46]. The Kruskal-Wallis and Mann Whitney tests (non parametric approaches) were used to compare target quadrant preferences during the probe trials. Cytokine expression was also analyzed using the Kruskal-Wallis test and post hoc Mann Whitney tests.

## Results

### Investigation of repeated methamphetamine exposure and RTL treatment on cognitive function

Mice exposed to methamphetamine or RTL treatment did not show any serious adverse effects or overt signs of ill health; Table S1 summarizes body weight information for the experiments.

Statistical analyses revealed no significant treatment group differences across study groups in terms of the latency to find the platform during the visible platform training sessions [Days 1 and 2; p = 0.39 (Gaussian approximation)] (Fig. 1A). This demonstrated that the groups were not significantly different in terms of basic motor function and perceptual abilities after treatment. Similar analyses also revealed no significant differences across treatment groups in terms of latency to find the platform during the hidden platform sessions [Days 3–5; p = 0.39 (Gaussian approximation)], suggesting that all groups were able to learn the task.

Methamphetamine exposure induced significant spatial memory impairments in mice. Immunotherapy with RTL significantly attenuated methamphetamine induced spatial memory impairments in mice as measured by quadrant preference during the final probe trial (*i.e.*, 24 hours after the completion of the Intervention Phase) (Fig. 1B). Friedman and Dunn post hoc tests revealed the following differences between the target and the three non-target quadrants: 1) Sal+Veh: Target versus Quadrants 1, 2, and 3 (p<0.05), 2) Sal+RTL551: Target versus Quadrants 1 (p<0.05), 2 (p<0.01), and 3 (p<0.05), and 3) Meth+RTL551: Target versus Quadrants 1 and 2 (p<0.05). In the Meth+Veh group, the percent time spent in each of the quadrants was not statistically different across quadrants, indicating that methamphetamine induced significant memory impairments in this group. However, in the Meth+RTL551 group, mice spent significantly more time in the target quadrant than in two out of the three other quadrants, indicating that RTL significantly attenuated the methamphetamine induced spatial memory impairments in this group.

### Evaluation of binge methamphetamine exposure and RTL treatment on cognitive function and cytokine expression

A neurotoxic, binge-level methamphetamine exposure model was used to more comprehensively model the human experience with methamphetamine and ultimately test the efficacy of RTL in reducing the more enduring methamphetamine-induced cognitive deficits [48], [51]. Consistent with our previous results, methamphetamine exposure induced significant spatial memory impairments in mice. Kruskal-Wallis tests revealed no significant treatment group differences across study groups in terms of the latency to find the platform during the visible platform training sessions [Days 1 and 2; p = 0.39 (Gaussian approximation)] (Fig. 2A). This demonstrated that the groups were not significantly different in terms of basic motor function and perceptual abilities after treatment. Similar analyses also revealed no significant differences across treatment groups in terms of latency to find the platform during the hidden platform sessions [Days 3–5; p = 0.39 (Gaussian approximation)], suggesting that all groups were able to learn the task.

Immunotherapy with RTL significantly attenuated methamphetamine induced spatial memory impairments in mice as measured by quadrant preference during the Day 5 probe trial (*i.e.*, the last day of hidden platform training) (Fig. 2B). Statistical analyses revealed significant differences between the target and the three non-target quadrants for the following treatment groups: 1) Sal+Veh: Target versus Quadrants 1 (p<0.001), 2 (p<0.01) and 3 (p<0.05), 2) Meth+Veh: Target versus Quadrant 1 (p<0.001), 3) Sal+RTL551: Target versus Quadrants 1 (p<0.05), 2 (p<0.01), and 3 (p<0.01), 4) Meth+RTL551: Target versus Quadrants 1 (p<0.001), 2 (p<0.01) and 3 (p<0.05). In the Meth+Veh group, the percent time spent in each of the quadrants was only statistically different between quadrant 1 and the target quadrant, indicating that methamphetamine had induced significant memory impairments in this group. However, in the Meth+RTL551 group, mice spent significantly more time in the target quadrant than in all three of the other quadrants, indicating that RTL had significantly attenuated the methamphetamine induced spatial memory impairments in this group.

To evaluate changes in CNS cytokine levels, hypothalamic samples were used for the multiplex detection of IFN-γ, IL-10, IL-1β, IL-2, IL-6, MCP-1, and TNF-α. Cytokine differences across treatment groups were analyzed using Kruskal-Wallis tests, and IL-2 was the only cytokine to show a significant effect of treatment (p = 0.028) (Table S2). Post hoc tests indicated that the Meth+Veh group had significantly higher levels of IL-2, as compared with the Sal+Veh (p = 0.007) and Meth+RTL551 (p = 0.032) groups.

## Discussion

This study evaluated a partial MHC/neuroantigen peptide construct (RTL551; pI-A^b^/mMOG-35-55), as a novel treatment strategy for methamphetamine induced cognitive impairments. The results of these experiments demonstrated that RTL551 has a treatment effect for memory impairments induced by repeated methamphetamine exposure. This treatment effect was demonstrated in two different mouse models of methamphetamine addiction (*i.e.*, 4 mg/kg/day and 40 mg/kg/day). We initially evaluated the efficacy of RTL551 when administered during continued methamphetamine exposure; humans frequently relapse during addiction treatment [45], so it is important to understand how treatments impact outcomes in a model of relapse and active use. We subsequently evaluated the efficacy of RTL551 administered during remission from repeated binge methamphetamine exposure; this treatment regimen closely models an optimal intervention strategy in humans where the goal is to treat persistent neuropsychiatric symptoms during remission from methamphetamine dependence. In all experiments, RTL551 successfully attenuated methamphetamine induced memory impairments in mice. Moreover, mice tolerated both methamphetamine exposure models and RTL551 treatment without apparent difficulty.

Treatment with partial MHC class II constructs (also referred to as recombinant T-cell receptor ligands - RTL) linked to antigenic peptides inhibit recruitment of inflammatory cells to brain [38]. It was recently reported that a novel regulatory pathway that involves RTL binding to CD11b(+) mononuclear cells through a receptor comprised of MHC class II invariant chain (CD74), cell-surface histones, and MHC class II may be contributing to its therapeutic effects [52]. Binding of RTL constructs with CD74 appears to involve MHC class II-α1/CD74 interactions that inhibit CD74 expression, block activity of its ligand, macrophage migration inhibitory factor (MIF), and reduce inflammation [52]. MIF is a product of the anterior pituitary gland and has been described as: 1) a pituitary peptide released during the physiological stress response, 2) a pro-inflammatory cytokine secreted after lipopolysaccharide (LPS) stimulation, and 3) a T cell product expressed as part of the antigen-dependent immune response. MIF also antagonizes glucocorticoid inhibition of T-cell proliferation by restoring IL-2 and IFN-γ production [53], [54].

Therefore, as an initial step toward understanding how RTL551 may impact brain function in the context of methamphetamine use, we measured the expression of pro- and anti-inflammatory cytokines in the hypothalamus following exposure to methamphetamine (or saline) and treatment with RTL551 (or vehicle). The hypothalamic-pituitary-adrenal (HPA) axis plays a critical role in the control of inflammation, and the hypothalamus, in particular, is a key brain region for CNS-to-periphery interactions and one that shows significant damage and altered functioning following methamphetamine exposure [55]–[57], including morphological changes and enhanced cortisol secretion that can contribute to the persistent neuropsychiatric impairments following methamphetamine use [56]. RTL inhibition of MIF signaling may help to reduce inflammation following methamphetamine exposure (and other brain insults). Consistent with this hypothesis, we found that levels of IL-2 (a potent T cell growth factor) were significantly higher in hypothalamic samples from methamphetamine exposed mice treated with vehicle, as compared with: 1) saline exposed mice, and 2) methamphetamine exposed mice treated with RTL551, suggesting that RTL551 may have contributed to a reduction in hypothalamic IL-2 levels in mice exposed to methamphetamine.

In experimental models of other neuroinflammatory conditions such as multiple sclerosis and stroke, pMHC/neuroantigen peptide constructs have been shown to successfully: 1) repair myelin and axonal damage back to baseline levels prior to disease induction, 2) reduce numbers of infiltrating inflammatory cells in the central nervous system, 3) reduce expression of intracellular adhesion molecule and vascular adhesion molecule on vascular endothelial cells, likely contributing to the reduction in infiltrating inflammatory cells, 4) reduce levels of inflammatory cytokines, chemokines, and chemokine receptors, 5) reduce infarct size following middle cerebral artery occlusion, and 6) prevent or reverse clinical disease signs [38], [39], [40], [41]. These findings and our own results suggest that pMHC/neuroantigen peptide constructs may help heal the brain following addiction and treat the immunological and neuropsychiatric impairments that persist for humans during remission from addiction [37].

To date, pharmacotherapeutic development for stimulant addiction has primarily focused on: 1) dopamine reuptake inhibitors (*e.g.*, bupropion) and other dopaminergic agents to chemically block the reward associated with drug taking, 2) antidepressants, anxiolitics, or antipsychotics to reduce psychiatric symptoms such as depression or anxiety during withdrawal and early remission from addiction, and 3) medications [*e.g.*, ondansetron, a serotonin receptor antagonist (5-HT_3_)] to reduce cravings and withdrawal symptoms. A recent review added to this list by summarizing the literature on cognitive enhancers which primarily target the cholinergic and noradrenergic systems [58]. One key drawback of neurotransmitter based therapies is that alone they do not offer a mechanism for *repairing* stimulant-induced neuronal injury—which might be vital for successful recovery. Agents specifically designed to repair neuronal damage may have increased potential to help adults regain lost function (*i.e.*, improved cognition and mood), re-engage in meaningful work and relationships, and avoid relapse [37]. Unfortunately, the results from clinical trials continue to be disappointing, with only marginally improved outcomes and high relapse rates [6], [59], [60]. Further, long-term use of many of these medications may not be optimal given their abuse potential (*e.g.*, methylphenidate, modafinil [61]).

## Conclusions

Our initial experiments suggest that a partial MHC/neuroantigen peptide construct (RTL551) can improve cognitive functioning and may also reduce IL-2 levels in the hypothalamus in mice exposed to methamphetamine. These results indicate that neuroimmune targeted therapies, and specifically MOG based RTLs, may have potential as treatments for methamphetamine-induced neuropsychiatric impairments.

Limitations of this study included the use of only one behavioral task, a single dose strength for RTL, and relatively small sample sizes. Future studies may also examine the effects of RTL treatment on other methamphetamine induced psychiatric impairments (*e.g.*, depression and anxiety) and addictive behaviors (*e.g.*, drug-seeking and relapse).

## Supporting Information

Table S1 **Body weight information.**
(DOCX)

Table S2 **Cytokine expression in the hypothalamus following methamphetamine exposure and RTL treatment.**
(DOCX)
