# Supplementary material for: Partial MHC/Neuroantigen Peptide Constructs: A Potential Neuroimmune-Based Treatment for Methamphetamine Addiction
Source: PLoS One. 2013 Feb 27;8(2):e56306. doi: 10.1371/journal.pone.0056306 (PMC3584080; doi:10.1371/journal.pone.0056306)
Supplement: Table S1 — Body weight information. (DOCX) [file pone.0056306.s001.docx]

**Supplementary Table 1** Body weight information

| *Investigation of repeated methamphetamine exposure and RTL treatment on cognitive function* | | |
| --- | --- | --- |
| **Treatment groups** | **Drug Exposure Phase^a^** | **Intervention Phase^a^** |
| Meth + Veh (n=8) | 24.4 (0.4) | 24.6 (0.4) |
| Meth + RTL551 (n=8) | 23.9 (0.2) | 24.2 (0.4) |
| Sal + Veh (n=8) | 24.3 (0.3) | 24.3 (0.2) |
| Sal + RTL551 (n=8) | 24.2 (0.3) | 24.4 (0.2) |
| *Evaluation of binge methamphetamine exposure and RTL treatment on cognitive function* | | |
| **Treatment groups** | **Drug Exposure Phase^a^** | **Intervention Phase^a^** |
| Cohort 1 |  |  |
| Meth + Veh (n=8) | 27.42 (1.42) | 27.98 (1.88) |
| Meth + RTL551 (n=8) | 29.44 (1.63) | 29.08 (1.50) |
| Sal + Veh (n=8) | 27.32 (1.44) | 27.68 (1.66) |
| Sal + RTL551 (n=8) | 26.97 (1.69) | 27.17 (1.47) |
| Cohort 2 |  |  |
| Meth + Veh (n=8) | 25.65 (0.45) | 25.91 (0.65) |
| Meth + RTL551 (n=8) | 26.36 (0.50) | 26.55 (0.75) |
| Sal + Veh (n=8) | 25.44 (0.48) | 25.98 (0.50) |
| Sal + RTL551 (n=8) | 25.79 (0.56) | 25.80 (0.57) |

^a^Data shown are mean body weights in grams (standard deviation).
