# Supplementary material for: Partial MHC/Neuroantigen Peptide Constructs: A Potential Neuroimmune-Based Treatment for Methamphetamine Addiction
Source: PLoS One. 2013 Feb 27;8(2):e56306. doi: 10.1371/journal.pone.0056306 (PMC3584080; doi:10.1371/journal.pone.0056306)
Supplement: Table S2 — Cytokine expression in the hypothalamus following methamphetamine exposure and RTL treatment. (DOCX) [file pone.0056306.s002.docx]

**Supplementary Table 2** Cytokine expression in the hypothalamus following methamphetamine exposure and RTL treatment.

| **Cytokines^a^** | **Sal + Veh** | **Sal + RTL551** | **Meth + Veh** | **Meth + RTL551** |
| --- | --- | --- | --- | --- |
| IFN-γ | 7.83 (2.68) | 4.51 (2.51) | 6.99 (1.40) | 6.03 (1.58) |
| IL-1β | 13.92 (3.68) | 15.50 (3.58) | 15.06 (2.67) | 13.81 (2.13) |
| **IL-2** | **15.77 (0.56)** | **17.84 (2.87)** | **19.40 (2.34)** | **15.91 (1.04)^b^** |
| IL-6 | 0.50 (0.29) | 0.58 (0.27) | 0.59 (0.29) | 0.46 (0.14) |
| IL-10 | 3.29 (0.96) | 3.53 (1.04) | 4.19 (1.26) | 2.59 (1.13) |
| MCP-1 | 47.86 (12.40) | 38.31 (18.38) | 53.84 (5.45) | 42.08 (6.81) |
| TNF-α | 1.30 (0.74) | 2.43 (1.25) | 2.98 (1.27) | 1.61 (0.66) |

^a^Values shown are mean pg/ml (standard deviation).  ^b^Cytokine differences across treatment groups were analyzed using Kruskal-Wallis tests, and IL-2 was the only cytokine to show a significant effect of treatment (p = 0.028). Post hoc tests indicated that the Meth + Veh group had significantly higher levels of IL-2, as compared with the Sal + Veh (p = 0.007) and Meth + RTL551 (p = 0.032) groups.
